# Can an Educational Handout Enhance Placebo Analgesia for Experimentally-Induced Pain?

**DOI:** 10.1371/journal.pone.0077544

**Published:** 2013-10-25

**Authors:** Chi Wang Tang, Ben Colagiuri

**Affiliations:** 1 School of Psychology, University of New South Wales, Sydney, Australia; 2 School of Psychology, University of Sydney, Sydney, Australia; University of Louisville, United States of America

## Abstract

The placebo effect is an interesting phenomenon whereby a dummy treatment can produce therapeutic benefit, such as, pain relief. While evidence for the placebo effect is growing, relatively few studies have explored ways of enhancing placebo effects. To address this, the current study tested whether placebo-induced analgesia could be enhanced by providing an educational handout about the efficacy of analgesics. Fifty university students were allocated to receive placebo treatment under the guise of a new analgesic formula, either with or without an educational handout, or to a no treatment control group before undergoing electrical and cold pressor pain tests. There was a placebo effect for electrically-induced pain with those receiving placebo treatment reporting significantly less pain compared with those who received no treatment. There was also some evidence of enhancement of this placebo-induced analgesia for electrically-induced pain as a result of the educational handout. No differences were found on cold pressor-induced pain. These findings suggest that providing educational information about a treatment could enhance its efficacy via the placebo effect. Future studies should test different methods of providing educational information in order to determine which elicit the strongest effects.

## Introduction

The placebo effect is a fascinating phenomenon in which therapeutic benefit can be derived from a dummy treatment. In the archetypical example, saline injections administered to wounded soldiers under the guise of morphine ameliorated their pain. Importantly, placebo effects can also enhance or impair responses to active treatments [1]. As such, determining if and how the placebo effect can be enhanced could prove beneficial by allowing health professionals to tailor treatments in order to maximize treatment outcomes via the placebo effect.

Before discussing evidence for and the characteristics of the placebo effect, it is worth noting that some authors have argued against the use of the term placebo effect in favour of terms such as the meaning response [2], context effects [3], and the care effect [4]. These approaches aim to highlight the fact that this phenomenon is not constrained to situations in which a dummy treatment, e.g. a sugar pill or saline injection, is administered. However, we do not consider the term placebo effect as being limited to such situations and prefer its use given that it is the term predominantly used throughout medicine and other related areas. Similarly, while it is increasingly common to separate positive and negative effects into placebo and nocebo effects, respectively, we use the single term placebo effect to refer to all such effects. This is because, as with many medications and procedures, placebos can produce both beneficial and adverse effects simultaneously [5]. Therefore, defining these effects separately as placebo effects and nocebo effects would lead to the potentially confusing conclusion that a substance or procedure could be both a placebo and a nocebo at the same time [6], [7]. Further, it may not always be clear whether a response is positive or negative and some responses may even be neutral [7]. For example, placebo alcohol could cause a feeling of intoxication, which one person might experience as pleasant, while another person experiences it as unpleasant. This would lead to the unnecessarily complicated conclusion that placebo alcohol produced a placebo effect in the first person but a nocebo effect in the second person. Defining the placebo effect broadly as incorporating positive, negative, and neutral effects overcomes these problems.

Some remarkable placebo effects have been observed over the past two decades. Placebo surgery for osteoarthritis of the knee has proven just as effective as the real surgery [8]. Sham deep brain stimulation has been found to produce just as much improvement in Parkinson's disease symptoms as actual deep brain stimulation does [9]. Perhaps most interestingly, open placebo treatment, that is, when participants know they are receiving nothing more than a sugar pill, has been found to improve symptoms of both irritable bowel syndrome [10] and major depressive disorder [11]. Further, as a result of recent advances in neuroimaging, some of the brain mechanisms involved in producing placebo effects are beginning to be understood. For example, Bingel and colleagues [12] found that instruction-induced modulation of the analgesic remifentanil was associated with changes in activity of known pain and opioid-sensitive brain regions consistent with the direction of pain modulation.

These landmark studies are supported by a large body of experimental evidence in support of the placebo effect. Pain has been the most heavily studied condition, with numerous studies demonstrating that placebo treatment can produce pain relief [13]–[17]. Although less extensively studied, there is also evidence for placebo effects across a range of other clinical and experimental settings. For example, placebo administration has been found to reduce depressive symptoms [18], [19], improve motor performance in Parkinson's disease [9], reduce sleep difficulty [20], [21], reduce smoking withdrawal symptoms [22], reduce seizure frequency in epilepsy [23], and improve cognitive performance [24], [25], as well as motor performance [26].

Expectancy is considered a key mechanism of the placebo effect [7]. Kirsch [27] has provided the most detailed account of how expectancies could generate placebo effects based on response expectancies. Response expectancies are anticipations of future involuntary states, such as, pain, arousal, and mood. Kirsch [27] argues that these response expectancies are self-fulfilling in that their activation generates the expected response. According to this view, a placebo manipulation elicits its effect by activating a response expectancy. In pain, for example, the administration of a placebo capsule, say, with the suggestion that it is a powerful analgesic activates the response expectancy for pain relief, which is sufficient to reduce pain in and of itself.

Given evidence of placebo effects across a range of conditions, an interesting question concerns if and how the placebo effect could be enhanced in order to maximize treatment outcomes. There is already evidence to suggest that the characteristics of a treatment can affect the strength of the placebo effect, presumably by strengthening the expectancy for an effect. Blue placebo capsules appear to produce stronger sedative effects than pink ones and yellow capsules were better than green or red capsules for treating depression [28]. A regimen of four placebo pills per day led to faster healing of duodenal ulcers than a regimen of two pills per day [29]. Placebo acupuncture reduced persistent arm pain more than placebo pills [30]. Further, simply being given a choice in treatment can enhance placebo-induced analgesia [31].

An alternative method of enhancing the placebo effect might be via providing additional information about the treatment. This has the benefit of being both cheap and flexible in comparison to adjusting characteristics of the treatment itself, such as the route of administration. Relatively few studies have tested whether additional information can enhance the placebo effect. In one study comparing real versus placebo acupuncture for experimentally-induced pain, participants were randomized to receive one of the two treatments with information that prior research indicated acupuncture was either effective, ineffective, or had variable efficacy [32]. In this study, acupuncture was only found to reduce pain when participants had been given information suggesting that it was effective.

In another study, first time chemotherapy patients were allocated to receive ondansetron, an antiemetic, either with educational information about its efficacy or with no additional information [33].While this information appeared to reduce the patients' expectancies for nausea, there was no actual reduction in their post-chemotherapy experience of nausea, suggesting a lack of any placebo enhancement. However, it is quite possible that no effect of the information was observed due to a floor effect caused by ondansetron's efficacy. Supporting this, almost half of the participants reported mild to no nausea as their peak nausea severity and the average nausea severity was only 1.76 and 1.86 out of 7 for the control and intervention group, respectively. Thus, it may not have been possible to observe an enhanced placebo effect because nausea was already heavily reduced.

To address this, the current study tested whether a written educational handout could enhance placebo analgesia for experimentally-induced pain. Using placebo, rather than active treatment should reduce the possibility of a floor effect masking the effect of the intervention. Based on previous research demonstrating placebo effects for pain [13]–[17], it was expected that placebo treatment would lead to reduced pain compared with no treatment. The question of primary interest was whether the educational handout could enhance this placebo effect.

## Methods

### Participants

Fifty participants (male  = 16; M_age_ = 22.24, SD  = 4.05) were recruited via advertisements, posted online at the University of New South Wales (UNSW) careers website, around the UNSW campus, and on a classified ads website (www.gumtree.com.au). Five participants of the total fifty were recruited from gumtree with the remainder recruited from UNSW. Potential participants who were lactose intolerant or allergic were excluded. All participants were given $40 reimbursement to cover out-of-pocket expenses associated with participating in the study.

### Design

Participants were randomly allocated to one of three groups: standard placebo, enhanced placebo, or no treatment control. Via an initial information sheet, all participants were led to believe that the study investigated the effects of a new formula painkiller, described as a combination of codeine and paracetamol, on pain. The standard placebo group was given a placebo capsule under the guise of this new analgesic with no additional information other than what was in the information sheet. The enhanced placebo group was given a placebo capsule along with a one-page educational handout with extra information detailing how analgesics work. Participants in the control group were given no treatment and no additional information. The dependent variables were pain ratings in response to electrical shock and both pain ratings and tolerance (time elapsed in seconds until hand withdrawal) on the cold pressor task. Gender was stratified across group by allocating males and females separately into the three conditions.

### Materials

#### Placebo Capsules

The placebo capsules were green and white coated (to resemble similar analgesics available in Australia) and contained approximately 250 mg lactose. They were prepared by the University of New South Wales pharmacy.

#### Educational Handout

The educational handout was a double-side sheet of paper detailing the mechanisms by which analgesics work. The handout suggested that the treatment was opioid-based and exerts its influence on the opioid system. This particular type of opioid-based analgesia explanation was chosen because the cover story mentioned codeine, which metabolizes into morphine, an opioid-based analgesic. Areas of the spinal cord and brain implicated were discussed and shown in a colored diagram and the explanation also focused on mu-opioid receptors, which is the main receptor that morphine acts upon.

#### Electrically-induced Pain

The shock apparatus consisted of two electrodes connected to the index finger of the participant's dominant hand. Isolated pulses were used to electrically induce pain, using PowerLab 8/36 and a Stimulus Isolator (ADInstruments Pty Ltd). For each shock, the pulse width was .2 ms with 10 repeats at 30 Hz. Calibration was carried out each session via stepwise increases of shock amplitude by 0.9 mA starting from 3 mA for each participant until he or she reported a pain rating of 7–8 out of 10, which was arbitrarily labeled 100% shock intensity for that participant. Each task involved three blocks of 3 shocks, on each at 60%, 80%, and 100% of the final calibration amplitude. Immediately following each shock, participants rated their pain on an 11-point scale ranging from 0 (No Pain), to 10 (Very Painful) presented via computer. On each trial, a warning signal, X, indicated a shock would occur in 10 sec, with an inter-trial interval of 15 sec.

#### Cold pressor

The cold pressor task involves submerging a hand in very cold water. In this study, participants were instructed to place their non-dominant hand, up to the wrist, in a 50 L cooler of ice water maintained at a temperature of approximately 5 degrees Celsius and instructed to “*try to keep it in for as long as possible*”. This temperature was selected to be within optimum range for observing the Lewis effect, in which pain is experienced due to vasoconstriction and subsequent vasodilation of blood vessels [34], [35]. An Aqua One Aquis 1000 canister filter was used to circulate the water to ensure consistent temperature throughout and to avoid the build-up of warm water around the participant's hand. The maximum time the participant kept their arm in the water (tolerance) was recorded, with an upper limit of 4 minutes. Also, pain levels at 20 s and 40 s and at tolerance were measured on an 11-point Likert-type scale ranging from 0 (No pain), to 10 (Intolerable pain) by asking the participants at the specific times “*How much pain do you feel now*?” During the task, to reduce possible demand characteristics, eye contact was avoided by having the back of the experimenter facing the participant and apparatus in the room.

#### Exit Questionnaire

An exit questionnaire was used as a manipulation check and to assess expectancies retrospectively. For the placebo groups, expectancy was assessed by asking the participants: “*How effective did you expect the analgesic you received in this study to be in terms of reducing your pain*?” on a 7-point scale ranging from 1 (Not effective) to 7 (Very effective). Manipulation for the enhanced placebo group (given the educational handout) was checked by asking “*How familiar are you with how analgesics work*?” on a 7-point scale ranging from 1 (Not Familiar) to 7 (Very Familiar), and “*How do you think the analgesic in this study works? [Provide as much detail as possible]*” with the target phrase being “mu-opioid receptors” or “pain receptors”. The control group was assessed for prior experience with analgesics only.

### Procedure

Participants attended two sessions as follows:

**Session 1: Familiarization (approximately 30 mins).** The first session was a familiarization session aimed at introducing participants to the pain tests. On arrival, participants were given the information sheet and consent form, which explained that the study was testing the efficacy of a new combination formula of paracetamol and codeine for pain relief. After providing consent, participants underwent the electrical shock task (calibration then test) followed by the cold pressor task. At the end of the session, the next session was scheduled, ideally two days after the first session.

**Session 2: Test Session (approximately 1 hour).** The second session was the test session. Participants were first recalibrated to the shock apparatus, after which a baseline shock test was carried out. Following this, participants in the standard placebo group were given the placebo capsule and a verbal instruction (“This is a potent painkiller. It is a fast-acting painkiller so we will allow 30 minutes for it to take effect”). Participants in the enhanced placebo group were given identical capsules and verbal instruction but also received the educational handout and asked to read it carefully. The control group was not given any treatment and was only instructed that the study would resume in 30 minutes. This aimed to minimize the impact of participants realizing that they were not going to receive any treatment. During the rest period, participants were provided with magazines and encouraged to read quietly. After 30 min from treatment, participants completed a posttreatment version of the electrical shock task (without recalibration). After this, all participants underwent the posttreatment cold pressor trial. At the end of the session, participants completed the exit questionnaire. The UNSW Human Research Ethics Committee approved all procedures.

### Statistical Analysis

One participant was removed from the data analysis due to their request for to be put in the control group due to an inability to ingest the pill for religious reasons. Planned orthogonal contrasts tested differences in posttreatment pain for placebo (standard and enhanced groups) versus no treatment and standard placebo versus enhanced placebo, controlling for baseline scores. For electrically-induced pain, the three shock trials at each intensity were averaged and grouped into 60%, 80%, and 100% shock intensity. The pain ratings from the baseline test in the experimental session were used as a covariate for electrically-induced pain. For the cold pressor, pain ratings and tolerance from the familiarization session were used as covariates because there was no baseline test in the experimental session. Data from the exit questionnaires (previous effectiveness of analgesics, expectations of pain relief, perceived effectiveness, familiarity with analgesics) were compared across groups via one-way ANOVAs. Pearson correlational analysis was run for the exit questionnaire data and the difference scores between baseline and posttreatment for outcome variables demonstrating a placebo effect. These correlations were considered exploratory; hence no correction for multiple comparisons was made. All analyses were conducted via IBM Statistics (Version 20) and results were considered statistically significant if p<.05.

## Results

### Participant Characteristics

Participants' age and gender across groups are shown in Table 1. There were no significant differences in age between the three groups, F(2,49) = .212, p = .81, nor in gender, χ^2^(2, N = 50) = 0.23, p = .989.

**Table 1:** Means and standard deviations for participant characteristics.

			Group	
		Enhanced Placebo	Standard Placebo	Control
n		16	16	18
Age	Mean	22.75	21.81	22.17
	(SD)	(5.58)	(3.54)	(2.88)
Gender	Male	11	11	12
	Female	5	5	6

### Placebo Effect for Electrically-induced Pain

**Figure 1:**
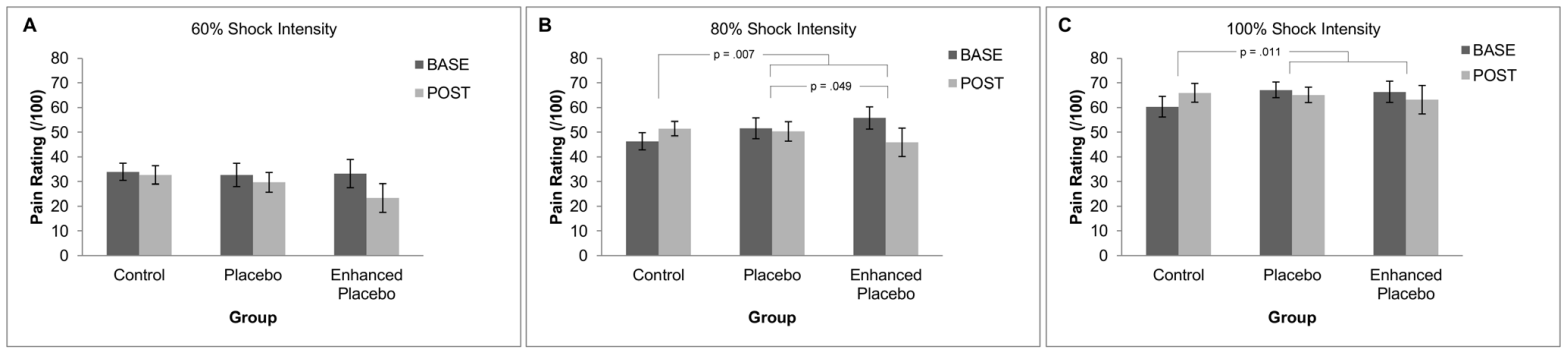
Mean (±SD) pain ratings pre- and posttreatment across groups for 60%, 80%, and 100% shock intensity in panel A, B, and C respectively. Placebo treatment significantly reduced pain at 80% and 100% shock intensities. Further, the enhanced placebo group had significantly less pain at 80% shock intensity than the standard placebo group.

Mean pain ratings at baseline and posttreatment across groups for electrically-induced pain are shown in Figure 1. As can be seen in Figure 1 (panel B), for the 80% shock intensity, placebo treatment significantly reduced pain relative to baseline by 1.08 (SD = 2.73) points compared with no treatment, F(1,46) = 8.07, p = .007, suggesting an overall placebo effect. Further, the enhanced placebo produced significantly greater pain relief of 9.85 (SD = 2.91) points than the standard placebo, F(1,46) = 4.08, p = .049, suggesting an enhanced placebo effect at this shock intensity. There was also an overall placebo effect at 100% shock intensity, with placebo treatment producing 3.02 (SD = 2.55) points less pain compared with no treatment, F(1,46) = 7.07, p = .011. However, there was no enhanced placebo effect at this shock intensity, F(1,46) = .019, p = .891. There were no statistically significant differences for either comparison at 60% shock intensity: overall placebo effect F(1,46) = .484, p = .490 and enhanced placebo effect F(1,46) = .009, p = .923.

### Placebo Effect for Cold Pressor-induced Pain

Mean pain ratings at baseline and posttreatment across groups for cold pressor-induced pain are shown in Table 2. The planned contrast analysis controlling for baseline ratings revealed no significant differences in posttreatment pain ratings when comparing placebo treatment with no treatment nor standard placebo with enhanced placebo at either 20 seconds or 40 seconds, all F<1. The same was true of tolerance, with no differences in tolerance between placebo treatment and not treatment, nor between standard placebo and enhanced placebo, highest F(1,46) = 1.54, p = .220. Thus, there was no placebo effect evident for cold pressor-induced pain.

**Table 2:** Mean (SD) for cold pressor pain ratings at 20 seconds, 40 seconds, and time to tolerance.

	Group					
	Enhanced Placebo		Standard Placebo		Control	
	Baseline	Posttreatment	Baseline	Posttreatment	Baseline	Posttreatment
Pain at 20 Seconds	6.13	6.47	6.20	6.20	6.97	7.06
(SD)	(2.67)	(2.75)	(2.02)	(2.48)	(1.87)	(2.16)
Pain at 40 Seconds	7.97	8.00	7.77	7.57	8.24	8.32
(SD)	(2.81)	(1.89)	(1.73)	(2.25)	(1.68)	(1.97)
Tolerance in seconds	139.4	141.9	138	160.4	99.2	108.7
(SD)	(101.1)	(97.1)	(97.0)	(99.0)	(93.3)	(97.1)

No differences were statistically significant

### Relationship between Expectancy, Prior Experience, and the Placebo Effect

Correlational analysis was conducted to investigate the relationships between expectancy, analgesic experience of the placebo, and the placebo effect itself. This analysis was restricted to the two outcomes for which there was evidence of a placebo effect, namely, 80% and 100% shock (calculated as a difference score of baseline minus posttreatment pain ratings). The relevant correlations are presented in Table 3. A statistically significant moderate correlation was found between how effective the treatment was expected to be and the 100% shock difference score (r = .445, p = .011), suggesting that a higher reported expected efficacy was associated with the placebo effect observed in the 100% shock pain ratings. Also, a significant moderate correlation was found between how effective the treatment was expected to be and how effective the treatment was perceived to be (r = .488, p = .005), suggesting higher reported expected efficacy was associated with higher reported efficacy. Interestingly, a significant negative correlation was found between prior experience of analgesics and the 80% shock difference score (r = −.294, p = .043), suggesting that a higher reported prior experience with analgesics was associated with a smaller placebo effect observed in the 80% shock pain ratings. No other correlations were statistically significant.

**Table 3:** Means and standard deviations for prior experience, expectations, perceived efficacy, and familiarity with analgesics.

	Group		
	Enhanced Placebo	Standard Placebo	Control
Prior Experience	4.53	5.13	4.53
(SD)	(1.06)	(1.36)	(1.40)
Expectation	4.75	3.94	-
(SD)	(1.73)	(1.88)	
Perceived Efficacy	3.75	3.44	-
(SD)	(1.65)	(1.83)	
Familiarity	3.06	3.63	-
(SD)	(1.39)	(1.82)	

### Manipulation checks

Table 4 shows participants' ratings for previous experience with analgesics, familiarity with analgesics, expectations of effectiveness (placebo groups only), and ratings of perceived effectiveness (placebo groups only) across groups. The enhanced placebo group reported a mean expectation of pain relief of 4.75 (SD = 1.73) whereas the standard placebo group reported a mean expectation of 3.94 (SD = 1.88). However, this did not reach statistical significance, F(1, 31) = 1.618, p = .213, nor did any other of these comparison, highest F(2, 49) = 1.13, p = .332.

**Table 4:** Correlational analyses between expectancy, analgesic experience, and placebo effect (as difference scores) for 80% and 100% shock.

	1	2	3	4	5	6
1. Prior Experience	1	.288	.102	−.135	−.294*	−.093
2. Expectation		1	.488**	.024	.251	.445*
3. Perceived Efficacy			1	.005	.366*	.432*
4. Familiarity				1	−.119	−.149
5. 80% shock					1	.568**
6. 100% shock						1

* . Correlation is significant at the 0.05 level (2-tailed).

** . Correlation is significant at the 0.01 level (2-tailed).

## Discussion

The current study tested whether an educational handout could enhance placebo analgesia for electrical and cold pressor-induced pain. As expected, there was a placebo effect for electrically-induced pain. Participants who were given placebo treatment reported significantly less pain at 80% and 100% shock intensity compared with those given no treatment. Importantly, this placebo-induced analgesia was enhanced at the 80% shock intensity in participants who received an educational handout about the efficacy of analgesics along with the placebo treatment. Despite these effects for electrically-induced pain, there was no evidence of a placebo effect of any kind for cold pressor-induced pain.

In terms of the overall placebo effect for electrically-induced pain, this is consistent with the previous studies demonstrating placebo treatment in the form of inert creams [13], [14], saline injections [36], and dummy electrodes [37] can produce pain relief. The current study extends these findings in the sense that to our knowledge, it is the first to demonstrate a placebo effect for electrically-induced pain with an oral placebo. Others have shown placebo effects with oral placebos for other types of pain, e.g. cold pressor-induced pain [31], [32] and heat-induced pain [17], [38]. Electrically-induced pain is a particularly useful model for exploring the placebo effect in the laboratory because stimulation can be repeated multiple times at the same site within a single session without damage to the skin, unlike the cold pressor and some types of heat-induced pain.

In terms of the enhanced placebo effect for those given the educational handout at 80% shock intensity, the current results differ from those of Shelke et al. [33] in that unlike them, we did find some evidence to suggest that educational information can enhance the placebo effect. Of course, the treatment settings across their study and the current one were quite different. The current study involved healthy volunteers undergoing experimentally-induced pain, whereas Shelke et al. 's [33] study involved a clinical sample of chemotherapy patients receiving prophylactic anti-emetics. Thus, the differences in findings could be due to factors such as the severity of the condition, the length of treatment, or whether or not an active treatment is delivered. The latter is particularly interesting as it relates to how placebo effects interact with active treatments. Placebo treatment was chosen in the current study because it provides a pure estimate of the placebo effect. However, it is possible that the magnitude and characteristics of the placebo effect may differ depending on whether a placebo treatment is administered (as in the current study) or an active treatment is administered (as in Shelke et al [33]). This is because, as we have argued previously, the placebo effect may not simply add onto the active treatment effect [24], [39]. That is, placebo and active treatment effects may be interactive, rather than additive.

Nonetheless it is clear from numerous studies that placebo interventions can enhance the response to an active treatment [12], [31], [40]. Thus, regardless of whether or not placebo and active treatments are additive in the true sense of the word, there does appear to be a placebo component to many treatments. Importantly, the current findings suggest that educational handouts may enhance this placebo component thereby enhancing the overall therapeutic effect for at least some conditions. To the extent that the current findings do generalize to clinical practice, they suggest that improved therapeutic outcomes could be achieved fairly cheaply by providing patients with additional educational information about the treatment they are receiving.

It was also interesting to note a positive and significant correlation between expectancy and pain relief at the 100% shock intensity. Although not statistically significant, the direction of the correlation for expectancy and pain relief at 80% was consistent with this. Taken with the tendency for stronger expectancies in the enhanced placebo group compared with the standard placebo group, this suggests that the educational manipulation and the general placebo effect were likely mediated via expectancy, as suggested by Kirsch [27]. Of course, caution is required here given that this interpretation is based on trends in the descriptive data, rather than statistically significant relationships. The only way to test whether these relationships hold would be to extend the current findings to substantially larger samples.

One potential limitation to the current study was the lack of a placebo effect at 60% shock intensity and on the cold pressor outcomes. A possible explanation for the lack of effect at 60% shock intensity is that the low level of stimulation may have led to a floor effect whereby a reduction in pain due to placebo treatment was unable to be observed, as may have been the case in Shelke et al. [33] described above. The lack of placebo effect for the cold pressor task may also not be surprising. A number of previous studies have also failed to find a placebo analgesic effect for this type of pain. For example, Knox and colleagues [32] found no effect of placebo acupuncture on cold pressor-induced pain. Similarly, Rose and colleagues [31] found that a placebo treatment only reduced cold pressor pain when participants were given a choice of two placebo treatments, but not when they were given no choice as in the current study. Furthermore, in the current study, the cold pressor test took place after the electrical shock test and this may have interfered with a possible placebo effect on the former. In general, there was high variance in both pain ratings and tolerance on the cold pressor task. As such, if a placebo effect does exist for the cold pressor task, then substantially larger samples may be required to demonstrate a placebo effect for this type of pain than were included here and in similar studies.

Overall, the present study found evidence for placebo-induced analgesia using placebo capsules in electrically-induced pain, but not cold pressor pain. The study also found some support for the possibility of enhancing placebo-induced analgesia by enhancing expectancies via an educational handout. These findings suggest that providing additional information about a treatment may enhance the placebo effect and improve therapeutic outcomes in at least some conditions. Future research could explore whether certain types of information delivery, including practitioner-patient communication, produce greater improvement via the placebo effect than others, with the aim of incorporating these procedures into clinical practice where effective.
